# Improving the Quality of SHO Seclusion Review Documentation: A Quality Improvement Project

**DOI:** 10.1192/bjo.2026.11515

**Published:** 2026-06-30

**Authors:** Robyn Wilcha, Megan Viegas, Lucy Morris, Ellen McCloy-Smith, Darren Bell

**Affiliations:** South West London & St George’s Mental Health NHS Trust, London, United Kingdom

## Abstract

**Aims:**

The aim of this quality improvement project was for initial seclusion reviews completed by resident doctors to achieve 80% compliance when standardised against all domains featured in the Mental Health Units (Use of Force) Act 2018 between the dates of September 2025 and January 2026.

**Methods:**

A quality improvement project was undertaken between September 2025 and January 2026. A driver diagram was developed at project initiation to identify primary drivers, which included improving clinicians’ understanding of documentation requirements, increasing access to relevant criteria and resources and enhancing multidisciplinary knowledge regarding patient seclusions. Secondary drivers included targeted education for new and current doctors, improving accessibility and usability of the seclusion review template and policy and strengthening multidisciplinary team communication. A fishbone diagram was created, highlighting contributing factors related to people (time pressures, burnout, unfamiliarity with the process), process (time-consuming steps, inadequate handovers, limited out-of-hours support) and environment (reviews occurring overnight, limited software support). Based on these diagrams, a total of 4 PDSA cycles were performed, implementing 4 change ideas: (1) increasing accessibility to the seclusion proforma in handover, (2) circulating the proforma via email, (3) incorporating teaching into the academic programme, and (4) providing core trainee teaching in small groups. Regular audits were undertaken at two weekly intervals, and data was analysed using a Statistical Process Control chart.

**Results:**

Despite the interventions, there was minimal change in the overall compliance rate. Of the reviews that met all required criteria, compliance increased from 75% to 83%. This is likely attributable to the small number of seclusion reviews occurring within each two-week period, as well as the random variation introduced by different on-call doctors. These factors produced substantial fluctuations in the results, depending on the doctors' level of training and the workload of their shifts. At the level of individual domains, documentation of risk assessment to self and others alongside subjective and objective assessments of physical health increased, indicating a positive change.

**Conclusion:**

This quality improvement project demonstrates that achieving the target of 80% compliance with the seclusion review requirements set out in the Mental Health Units (Use of Force) Act 2018 is unlikely to be sustained through educational or individual-level interventions alone. More directive system-level approaches may be required for sustained compliance, such as mandatory completion of all domains prior to submission of a seclusion review and efforts to reduce the overall use of seclusion to allow adequate time for high-quality, reflective documentation. Improving compliance with seclusion reviews is central to patient safety and safeguarding the rights of service users.

